# Implicit Measures of Actual Versus Ideal Body Image: Relations with Self-Reported Body Dissatisfaction and Dieting Behaviors

**DOI:** 10.1007/s10608-018-9917-6

**Published:** 2018-05-16

**Authors:** Klaske A. Glashouwer, Elise C. Bennik, Peter J. de Jong, Adriaan Spruyt

**Affiliations:** 1 Department of Clinical Psychology and Experimental Psychopathology, University of Groningen, Grote Kruisstraat 2/1, 9712 TS Groningen, The Netherlands; 2 Department of Eating Disorders, Accare Child and Adolescent Psychiatry, Groningen, The Netherlands; 3 Department of Experimental-Clinical and Health Psychology, Ghent University, Ghent, Belgium

**Keywords:** Implicit body image, Body dissatisfaction, Relational responding task, Dieting

## Abstract

Body dissatisfaction refers to a negative appreciation of one’s own body stemming from a discrepancy between how one perceives his/her body (*actual body image*) and how he/she wants it to be (*ideal body image*). To circumvent the limitations of self-report measures of body image, measures were developed that allow for a distinction between actual and ideal body image at the implicit level. The first goal of the present study was to investigate whether self-reported body dissatisfaction is related to implicit measures of actual and ideal body image as captured by the Relational Responding Task (RRT). Secondly, we examined whether these RRT measures were related to several indices of dieting behavior. Women high in body dissatisfaction (*n* = 30) were characterized by relatively strong implicit I-am-fat beliefs, whereas their implicit I-want-to-be-thinner beliefs were similar to individuals low in body dissatisfaction (*n* = 37). Implicit body image beliefs showed no added value over explicit body image beliefs in predicting body dissatisfaction and dieting behavior. These findings support the idea that the interplay between ideal and actual body image drives (self-reported) body dissatisfaction. However, strong support for the view that it would be critical to differentiate between explicit and implicit body image beliefs is missing.

## Introduction

Body image attitudes are assumed to play a central role in organizing thoughts, behaviors, feelings, and evaluations related to one’s body (Cash 2002, 2011). Negative body image attitudes may manifest itself by a strong importance of as well as a negative appreciation of one’s shape and weight, i.e. body dissatisfaction. In Western societies, a significant part of the female population is characterized by a relatively high degree of body dissatisfaction (e.g., Tiggemann 2004). Body dissatisfaction is thought to stem from a discrepancy between how one perceives his/her body (*actual body image*; e.g., Cash 2011) and how he/she wants it to be (*ideal body image*, i.e., internalized ideals about one’s physical appearance; e.g., Strauman et al. 1991; Thompson and Stice 2001; Williamson et al. 1993). So far, measures of actual and ideal body image typically relied on self-report questionnaires, which are susceptible to the influence of social desirability and self-deception. Moreover, they are unsuited, by definition, to capture traces of past experiences that are introspectively unidentified (Greenwald and Banaji 1995). Implicit measures circumvent these limitations, because they are designed to capture the to-be-measured construct under automaticity conditions (De Houwer et al. 2009). Since body image is a sensitive subject, the use of implicit measurement procedures might add valuable information to existing self-report measures. Consequently, our first aim was to investigate the relation between implicit measures of actual and ideal body image and self-reported body dissatisfaction. Secondly, since body image attitudes are assumed to play a central role in how individuals respond to situations and events that threaten or challenge their body image (Cash 2011), we wanted to examine whether implicit actual and ideal body image beliefs were also related to behaviors that may potentially follow from body dissatisfaction, such as dieting. Considering the just described problems related to self-report questionnaires, both self-reported dietary restraint and food intake as well as a behavioral measure of dieting were included in the design.

In several studies it was already examined to what extent people associate the attributes thin and fat with either a positive or negative valence (e.g., Ahern et al. 2008; Ahern and Hetherington 2006; Vartanian et al. 2005). However, these prior implicit measurement procedures are limited by an inability to capture relational information. When measuring body image, not only the strength of the association between concepts matters (e.g., between “I” and “thin”), but also *how* concepts are related (e.g., there is a crucial difference between “I *am* thin” or “I *want to be* thin”). Recently two “relational” implicit measures have been developed which aim to capture how concepts are related at the implicit level: The Implicit Relational Assessment Procedure (IRAP; Barnes-Holmes et al. 2006) and the Relational Responding Task (RRT; De Houwer et al. 2015).

Only a few studies so far have used a relational measure to index implicit body image beliefs. An IRAP was administered to assess implicit I-am (e.g., thin or fat) and I-want-to-be (e.g., thin or fat) beliefs in a sample of first-year female students (Juarascio et al. 2011). Individuals whose IRAP scores were indicative of stronger implicit I-am-fat beliefs showed an increase in self-reported body dissatisfaction over an 8-month period (Juarascio et al. 2011). Another study used an IRAP to assess implicit pro-thin and anti-fat attitudes towards the self and others and showed that individuals with anorexia were characterized by stronger anti-fat attitudes towards the self than the non-clinical, matched comparison group (Parling et al. 2012). However, this latter IRAP did not index whether individuals considered *themselves* to be thin or fat, nor did this study index implicit ideal body image. In two recent studies, Heider et al. (2015, 2018) did distinguish between implicit actual and ideal body image as separate components in one sample using two IRAPs and two RRTs, respectively. In a RRT, individuals are asked to indicate quickly whether statements do or do not correspond with a certain response rule. The response rule changes between blocks of trials. The premise of the RRT is that the speed of responding is influenced by the degree to which the response rule is congruent with the personal (implicit) beliefs of the respondent. For example, for the RRT measuring actual body image, individuals categorized statements according to the rule “I am skinny” in one block and according to the rule “I am fat” in another block. Both studies showed that female students high in self-reported body dissatisfaction were faster to classify statements according to the response rules “I am fat/I want to be thinner” in comparison to the response rules “I am thin/I want to be fatter”. It may be noted, however, that implicit body image did not predict self-reported body dissatisfaction over and above self-report (“explicit”) measures of actual and ideal body image (Heider et al. 2015, 2018). In sum, studies conducted so far indicate that self-reported body dissatisfaction indeed seems to be related to implicit actual and ideal body image (Heider et al. 2015, 2018), and there is some preliminary evidence that implicit actual body image predicts changes in body dissatisfaction over time (Juarascio et al. 2011).

Since relational implicit measures are quite a new class of instruments, the first goal of this paper was to replicate the study of Heider et al. (2018) and to test the robustness of these prior findings. More specifically, we used two RRTs to index implicit actual and ideal body image beliefs in a sample of women with either high or low body dissatisfaction (cf. Heider et al. 2018). In addition, we wanted to investigate whether implicit measures of actual and ideal body image (and their interaction) are predictive of body dissatisfaction over and above explicit measures of actual and ideal body image (cf. Heider et al. 2018). Based on the findings reported by Heider et al. (2015, 2018), we hypothesized that (i) higher levels of self-reported body dissatisfaction are associated with higher levels of implicit I-am-fat beliefs and higher levels of implicit I-want-to-be-thinner beliefs, (ii) implicit actual and ideal body image measures do not predict self-reported body dissatisfaction over and above explicit measures of actual and ideal body image, and (iii) the relation between self-reported body dissatisfaction and implicit I-want-to-be-thinner beliefs is most pronounced in individuals who are characterized by relatively strong implicit I-am-fat beliefs (Heider et al. 2018).

In addition, we were interested in investigating behaviors that may potentially follow from body dissatisfaction. Body image attitudes are assumed to play a central role in how individuals respond to situations and events that threaten or challenge their body image (Cash 2011). “Appearance fixing” is a response or coping strategy in which individuals try to correct or change aspects of their appearance that they dislike. Dieting is an often-used form of appearance fixing and self-reported dieting behavior has already been shown to be robustly related to self-reported (explicit) body dissatisfaction (e.g., Stice 2002; Stice and Shaw 2002). In addition, dieting is considered a key factor in predicting the onset, maintenance, and relapse of eating disorders (Carter et al. 2004; Fairburn et al. 1993; Johnson and Wardle 2005; Neumark-Sztainer et al. 2006). A second aim of this study was to investigate whether the combined use of implicit and explicit measures of body image allows for an improved prediction of dieting behaviors. This approach was inspired by the idea that implicit and explicit cognitions guide different kinds of behaviors (e.g., Gawronski and Bodenhausen 2006; Wilson 2002). Whereas explicit cognitions are thought to guide controlled behavior, automatic cognitions are thought to be especially relevant in guiding spontaneous, impulsive behaviors (e.g., Egloff and Schmukle 2002; Huijding and de Jong 2006; Spalding and Hardin 1999). Hence, to the extent that both types of processes contribute to dieting behavior, both implicit and explicit measures are needed to achieve accurate prediction. Dietary restraint was measured not only with a questionnaire, but also with a food diary to index actual food intake. In addition, as an index of spontaneous food selection, we included a behavioral task in which participants could choose between high caloric (candy bar) and low caloric (piece of fruit) foods. We hypothesized that stronger implicit I-am-fat beliefs and I-want-to-be-thinner beliefs would be related to more pronounced dietary restraints (i.e. more self-reported dieting behavior, less self-reported food intake, and more often choosing low caloric foods over high caloric foods), and that these relations would be most pronounced for the behavioral measure since implicit beliefs are thought to be especially relevant in guiding spontaneous behaviors.

## Method

### Participants

As part of an online survey consisting of several questionnaires, 235 first-year, female psychology students at the University of Groningen completed the 6-item Body Image States Scale (BISS; Cash et al. 2002) as an index of body dissatisfaction. The 25% highest and the 25% lowest scoring individuals were invited to participate in the current laboratory study. Both the online survey and the present study were presented as being part of the same research project about healthy lifestyle. An extreme groups approach was chosen to maximize the chance to detect meaningful inter-individual differences. The study sample consisted of 32 women high in body dissatisfaction (BISS: *M* = 6.45, *SD* = .85) and 40 women low in body dissatisfaction (BISS: *M* = 3.05, *SD* = .36). Sample sizes between groups differed, because more individuals from the satisfied group responded to the study invitation. Participants ranged in age from 18 to 25 years (*M* = 20.05; *SD* = 1.41). Students from both the Dutch and English educational psychology program were invited to participate, and they could choose whether they preferred the Dutch or English version of the questionnaires and computer tasks.

### Materials

#### Body Dissatisfaction

Body dissatisfaction was indexed with the 6-item Body Image States Scale (BISS; Cash et al. 2002). Items of the BISS are scored on a 9-point response scale and the mean score is used as an index of body dissatisfaction (range 1–9). In the current study, we chose to reverse the original scoring direction to facilitate interpretation of the findings: higher BISS scores indicate higher levels of body dissatisfaction. Reliability of the BISS in the current study was high (*α* = .90).

#### Relational Responding Tasks (RRT)

In line with the study by Heider et al. (2018), two RRTs were constructed to capture implicit beliefs about the actual body image (i.e., actual-RRT) and the ideal body image (i.e., ideal-RRT). A detailed description of the RRT and the trial procedure is given by De Houwer et al. (2015).

##### Stimuli

The trials of both RRTs consisted of inducer words and target statements. The inducer words were the same for both RRTs and consisted of five synonyms of ‘true’ and five synonyms of ‘not true’ (see Appendix Table 7). For each RRT, a set of 20 sentences was used as target statements (see Appendix Tables 8, 9). For the actual-RRT, five target statements were included about the belief to be thin (e.g., ‘I have a thin figure’) and five target statements about the belief to be overweight (e.g., ‘I weigh too much’). Ten additional target statements were formed by negations of the first ten statements (e.g., ‘I do not have a thin figure’ and ‘I do not weigh too much’). The purpose of the negation statements was to ensure that participants had to process the meaning of the entire statement (and not just a subset of words) to be able to respond correctly (Heider et al. 2018). For the ideal-RRT, five target statements were included about the desire to be thinner (e.g., ‘I want to have a thinner figure’) and five target statements about the desire to be more overweight (e.g., ‘I strive to weigh more’). Again, negations of these ten statements were included (e.g., ‘I want to have a less thin figure’ and ‘I do not strive to weigh more’).

##### Instructions and Design

In both RRTs, on each trial either an inducer word or target statement was presented on the computer screen. Participants were instructed to categorize the stimuli as ‘true’ or ‘not true’ by pressing the right or left ctrl-key of the keyboard, respectively. The purpose of the inducer trials was to prevent recoding of the responses, for example in terms of their physical location. Each RRT comprised five blocks (see Table 1 for an overview of the design). In Block 1, participants practiced the categorization of the inducer words. In Block 2, the first response rule was practiced, and participants were asked to respond, “as if they were skinny” (in the actual-RRT) or “as if they wanted to be skinnier” (in the ideal-RRT). In Blocks 3A and 3B, words and statements were mixed, i.e., the 10 inducer words were presented twice, and the 20 target statements were presented once. Participants were asked to respond in accordance with the response rules of Block 1 and 2. Block 4 was identical to Block 2, except that the response rule now was reversed. In this block, participants were asked to respond, “as if they were fat” (in the actual-RRT) or “as if they wanted to be fatter” (in the ideal-RRT). Blocks 5A and 5B were identical to blocks 3A and 3B, but participants were now asked to respond in line with the response rule for the statements practiced in Block 4. The response rule for the inducer trials remained the same throughout the entire experiment. The response labels ‘TRUE’ and ‘FALSE’ remained visible in the top-right and top-left corner of the computer screen throughout each block of trials. The response rules themselves were not presented during the actual completion of trials. The order of the stimuli was random within each block, with the restriction that the same stimulus could not be repeated on consecutive trials. Task performance in the RRT is thought to be the result of the degree to which a response rule is congruent with the personal (implicit) beliefs of the respondent. One can thus learn about the implicit beliefs of an individual by comparing mean response latencies between different blocks of trials (i.e., different rules).

**Table 1:** Design of the Relational Responding Task

Block	No. of trials	Description	Task
1	40	Practice inducer trials	Sort words
2	40	Practice response rule 1	Sort sentences according to rule 1
3A (Part 1)	40	Measurement rule 1	Mix of sorting words and sentences
3B (Part 2)	40	Measurement rule 1	Mix of sorting words and sentences
4	40	Practice response rule 1	Sort sentences according to rule 2
5A (Part 1)	40	Measurement rule 2	Mix of sorting words and reversed sentences
5B (Part 2)	40	Measurement rule 2	Mix of sorting words and reversed sentences

Rule 1: Respond as if you are skinny/want to be skinnier; Rule 2: Respond as if you are fat/want to be fatter. The sub-blocks (part 1 and part 2 of the same pair) were presented as a continuous stream of trials, which means that the difference between the sub-blocks was unnoticeable for the participants

Each trial started with the presentation of a stimulus (i.e., inducer word or target statement) in the middle of the screen, Tahoma, 28-point font size. In line with the original RRT developed by De Houwer et al. (2015), we manipulated the color of the stimuli to help participants switch between the inducer trials and the target trials. To minimize any overlap between the two RRTs, we used different colors for target trials of the actual-RRT and the ideal-RRT. Inducer stimuli were presented in white color in both RRTs, target statements were presented in orange (actual-RRT) or blue color (ideal-RRT). Stimuli were presented until the correct response was registered. Incorrect responses were signaled by the presentation of a red X (Arial, 72-point font size) below the stimulus until participants corrected their response. The next trial started 750 ms after registration of the correct response. The RRTs were presented on a 24-inch widescreen and were written in Affect 4.0 (Spruyt et al. 2010).

#### Explicit Actual and Ideal Body Image

Participants rated each of the 40 target statements used in the RRTs on a 5-point scale, ranging from 1 (completely disagree) to 5 (completely agree). The mean rating of the 20 target statements concerning actual body image was used as explicit measure of actual body image. Half of the items were reversed in scoring in a way that higher scores reflected stronger I-am-fat beliefs. The mean rating of the 20 target statements concerning ideal body image was used as explicit measure of ideal body image. Half of the items were reversed in scoring in a way that higher scores reflected stronger I-want-to-be-thinner beliefs. Reliability of both explicit measures was high (explicit actual: *α* = .97; explicit ideal: *α* = .91).

#### Self-Reported Restraint Eating

To index restraint eating, we used the subscale ‘restraint’ of the Eating Disorder Examination Questionnaire (EDE-Q 6.0; Fairburn and Beglin 2008). The EDE-Q is the questionnaire version of the Eating Disorder Examination interview and is used to assess eating disorder psychopathology during the last 28 days. The subscale ‘restraint’ consists of five items [e.g., “On how many of the past 28 days have you been deliberately trying to limit the amount of food you eat to influence your shape or weight (whether or not you have succeeded)?”] which are answered on a scale between 0 (no days) and 6 (every day). The EDE-Q has demonstrated good internal consistency, temporal stability, and reliability (Berg et al. 2012; Luce and Crowther 1999). In the current sample, the subscale restraint also showed a good internal consistency (*α* = .80).

#### Behavioral Measure of Food Selection

As a behavioral measure of food selection, participants were offered a plate filled with nicely arranged fruits and candy bars from which they could choose one item. The items were presented by the researcher at the end of the laboratory assessment and were offered under the guise of “a small gift for their participation”. Besides the researcher and the participant, no other people were present during the food selection. Two pieces of each fruit (an orange, an apple, and a banana) and each candy bar (a Snickers, a Mars, and a Kit-Kat) were offered. The fruits looked clean and fresh, and the candy bars were in their original packing. We kept track of whether the participant chose a candy bar.

#### Self-Reported Food Intake

Participants were asked to keep a food diary during 3 consecutive weekdays. We chose 3 instead of 5 weekdays, because we did not want to overburden the participants. With the help of a free online application (http://www.nutracheck.co.uk), they kept track of all foods and drinks consumed during this period. Afterwards they handed in an overview of their food intake. As an index of self-reported food intake, we calculated the mean number of consumed calories per day over these three days.

### Procedure

The study was approved by the Ethical Committee Psychology of the University of Groningen and informed consent was obtained from all individual participants included in the study. Participants received course credits in exchange for participation (*n* = 66). A few students already earned all the necessary course credits and took part in exchange for a monetary compensation of €15 (*n* = 6). During the online survey, participants completed the questionnaires (BISS, EDE-Q). Then, they were invited to the lab where both the actual-RRT and ideal-RRT were administered. Half of the participants started with the actual-RRT and the other half with the ideal-RRT (cf. Heider et al. 2018). Following both RRTs, participants gave explicit ratings of the RRT stimuli in a questionnaire administered via a computer. Then, we measured their length and weight to be able to calculate their body mass index (BMI = weight/length × length). Finally, participants were given the choice between a candy bar or a fruit as a “small gift for their participation”. Following the laboratory session, participants were asked to keep a food diary during 3 consecutive weekdays which they handed in a week later.

### Data Analyses

#### Data Reduction RRTs

In line with previous RRT research, the analyses were restricted to the data of the target trials (i.e., when target statements were presented) of the mixed blocks (blocks 3 and 5; see Table 1). Data of both the negated and non-negated target statements were included in the analyses. The data of three participants were excluded from the analyses because their mean reaction times in the actual-RRT and/or the ideal-RRT exceeded the cutoff criterion of 2.5 SDs above the grand mean of the task (actual-RRT *M* = 1504 ms, *SD* = 445 ms, threshold = 2617 ms; ideal-RRT: *M* = 1860 ms, *SD* = 503 ms, threshold = 3118 ms; cf. Heider et al. 2018). The data of two additional participants were excluded, because the error rates in the actual-RRT or the ideal-RRT exceeded the cutoff criterion of 2.5 *SD*s above the grand mean of the task (actual-RRT: *M* = 10.4%, *SD* = 5.9%, threshold = 25.1%; ideal-RRT: *M* = 11.8%, *SD* = 6.8%, threshold = 28.9%; cf. Heider et al. 2018). The final sample consisted of 30 women high in body dissatisfaction and 37 women low in body dissatisfaction.

For each RRT separately, raw response latencies were transformed into D-scores using the D-algorithm (D1^1^; Greenwald et al. 2003) which was originally developed for the IAT (cf. Heider et al. 2018). In accordance with the D1-algorithm, error latencies were replaced by the response latencies of the corrected responses and reaction times above 10,000 ms were discarded. The D-scores were calculated by subtracting mean reaction times of Block 5A from Block 3A (mixed block part 1; see Table 1) and Block 5B from Block 3B (mixed block part 2). These two difference scores were divided by the pooled standard deviations based on all responses in the specific blocks. Finally, for each RRT separately, the two resulting D-scores were averaged. For the actual-RRT, D-scores were computed such that higher scores reflected stronger implicit I-am-fat beliefs. For the ideal-RRT, D-scores were reversed such that higher scores indicated stronger implicit I-want-to-be-thinner beliefs to facilitate interpretation of the findings.

To examine the reliability of the RRTs, reliability coefficients were estimated using a bootstrap procedure (see also Heider et al. 2018). For each RRT and each of 1000 random splits of the data, the correlation across participants between the two RRT scores was calculated and spearman–brown corrected. These 1000 correlations were then averaged, which resulted in split-half reliability estimates of *R*sb = .45 and *R*sb = .49, for the actual-RRT and the ideal-RRT, respectively.

#### Statistical Analyses

To investigate whether the RRT scores were dependent upon the degree of self-reported body dissatisfaction, a 2 (body dissatisfaction: high vs. low) × 2 (RRT: actual vs. ideal) ANOVA was conducted with body dissatisfaction as between-subject factor and RRT type as within-subject factor. This analysis was repeated with explicit RRT type as within-subject factor to check the validity of the RRT target statements. In addition, we examined whether implicit and explicit measures of actual and ideal body image and their interaction were predictive of body dissatisfaction using hierarchical logistic regression analysis. In step 1, explicit ratings of actual and ideal body image were included as predictors. In step 2, we added implicit actual and ideal body image to the model. Finally, in step 3, the interaction between implicit actual and implicit ideal body image was added to the model. Next, to investigate whether implicit body image can be used to predict dieting behaviors over and above self-reported body image, two hierarchical regression analyses were conducted with EDE-Q restraint and self-reported food intake as dependent variables. Finally, a hierarchical logistic regression analysis was conducted with the behavioral measure (candy bar vs. no candy bar) as dependent variable. In the regression analyses, explicit actual and ideal RRT ratings were included as predictors in step 1 and actual and ideal RRT scores were added as predictors in step 2. Again, in step 3, the interaction between actual and ideal RRT scores was added to the model. We checked the data to make sure that assumptions were met for each specific analysis (e.g., normality of the residuals in case of regression analyses).

In addition, to increase the confidence in our results and test the evidence for the null-hypothesis in case of non-significant findings, we decided to complement the RM-ANOVA with a Bayesian approach. These post-hoc analyses were conducted using the free software JASP using default Cauchy priors (JASP Team 2017). To facilitate interpretation of the outcomes, BF_10_ (which quantifies the evidence for the alternative hypothesis over the null hypothesis, i.e., the groups differ), will be reported for those tests that provided significant results. BF_01_ (which quantifies the evidence for the null hypothesis over the alternative hypothesis, i.e., the groups do not differ) will be reported when the analyses showed insignificant findings. A BF higher than 3 is interpreted as moderate evidence, a BF between 10 and 30 is considered strong evidence, and a BF above 30 is interpreted as very strong evidence (Lee and Wagenmakers 2013; adjusted from; Jeffreys 1961).

## Results

### Descriptives

Explicit RRT ratings were missing for one participant and BMI was missing for another participant. In addition, data of self-reported food intake were missing for 11 participants. The group for which self-reported food intake was missing did not significantly differ from the rest of the sample with respect to BMI, implicit/explicit actual/ideal body image and EDE-Q restraint. However, the group for which measures of self-reported food intake were missing did show lower body dissatisfaction, 3.68 versus 4.73, *t* (19.52) = 2.32, *p* = .03, *d* = .67. Means and standard deviations per group are reported in Table 2. Individuals high and low in body dissatisfaction did not differ with respect to age. In addition, both groups significantly differed on all other variables except for implicit ideal body image.

**Table 2:** Means and standard deviations per group

	Total sample *n* = 67 *M (SD)*	High body dissatisfaction *n* = 30 *M (SD)*	Low body dissatisfaction *n* = 37 *M (SD)*	*t*-test *t (p)*
Age	20.05 (1.41)	20.11 (1.34)	20.00 (1.48)	.29 (.77)
BMI	22.72 (3.88)	25.25 (4.38)	20.74 (1.75)	5.23 (< .001)
BISS (1–9)	4.55 (1.78)	6.40 (.83)	3.06 (.38)	20.47 (< .001)
Actual-RRT	.02 (.31)	.13 (.28)	− .08 (.30)	2.99 (< .01)
Ideal-RRT	− .03 (.32)	− .10 (.34)	.03 (.29)	− 1.74 (.09)
Explicit actual (1–5)	2.68 (.97)	3.42 (.84)	2.11 (.60)	7.05 (< .001)
Explicit ideal (1–5)	3.81 (.62)	4.20 (.55)	3.50 (.48)	5.53 (< .001)
EDE-Q restraint (0–6)	1.10 (1.20)	1.82 (1.20)	.51 (.84)	5.04 (< .001)
Food intake (kcal)	1485.44 (490.84)	1309.52 (453.25)	1661.36 (470.28)	− 2.85 (< .01)
Behavioral measure^a^	70%	90%	54%	10.22 (< .01)^b^

*BMI* Body Mass Index, *BISS* Body Image State Scale, *RRT* Relational Responding Task, *EDE-Q* Eating Disorder Examination Questionnaire

^a^% choosing no candy bar

^b^Chi-square test was performed

The bivariate correlations between variables are shown in Table 3. Non-parametric Spearman’s correlations were calculated involving the BISS scores (group: high vs. low), EDE-Q restraint, and the behavioral measure, because these variables were not normally distributed. Actual-RRT scores were related positively to explicit actual body image, body dissatisfaction, and explicit ideal body image. These results suggest that relatively strong implicit beliefs are associated with relatively strong explicit I-am-fat beliefs, higher self-reported body dissatisfaction, and stronger explicit I-want-to-be-thinner beliefs. Ideal-RRT scores were not related to any other variables. Finally, in contrast to our expectation, neither actual-RRT nor ideal-RRT scores were significantly related to any of the dieting indices.

**Table 3:** Bivariate correlations between variables in the total sample

	1	2	3	4	5	6	7	8
1. Actual-RRT	–	.04	.52**	.42**	− .22			
2. Ideal-RRT		–	.00	.18	− .11			
3. Explicit actual			–	.69**	− .33*			
4. Explicit ideal				–	− .28*			
5. Self-reported food intake					–			
6. BISS^a^	.35**	− .20	.67**	.60**	− .35**	–		
7. EDE-Q restraint	.24†	.03	.58**	.72**	− .36**	.58**	–	
8. Behavioral measure^b^	.03	.01	.33**	.34**	− .14	.39**	.42**	–
9. BMI	.38**	− .03	.83**	.53**	− .33**	.61**	.44**	.33**

Correlational coefficients in the lower half are non-parametric correlations

*RRT* Relational Responding Task, *BISS* Body Image State Scale, *EDE*-*Q* Eating Disorder Examination Questionnaire

^†^*p* < .10; *p < .05; **p < .01

^a^Dichotomous measure: 0 = low body dissatisfaction; 1 = high body dissatisfaction

^b^Dichotomous measure: 0 = candy bar; 1 = no candy bar

### Is Self-Reported Body Dissatisfaction Related to Implicit Measures of Actual and Ideal Body Image?

A significant body dissatisfaction × RRT interaction was found, *F*(1, 65) = 12.52, *p* < .01, *η*^2^ = .16, BF_10_ = 83.91. The main effects of body dissatisfaction and RRT were not significant, *F*(1, 65) = .48, *p* = .49, *η*^2^ = .01, BF_01_ = 3.97 and *F*(1, 65) = 1.58, *p* = .21, *η*^2^ = .02, BF_01_ = 3.70 respectively. Post hoc *t*-tests showed that, on average, individuals high in body dissatisfaction scored higher on the actual-RRT than individuals low in body dissatisfaction, *t*(65) = 2.99, *p* < .01, *d* = .74, BF_10_ = 9.90. Thus, in comparison to individuals low in body dissatisfaction, individuals high in body dissatisfaction were, on average, faster in categorizing statements according to the rule “I am fat/I am not thin” than the rule “I am thin/I am not fat”. However, the two groups did not significantly differ from each other in terms of the ideal-RRT scores, *t*(65) = − 1.74, *p* = .09, *d* = .43, BF_01_ = 1.10. When BMI was included as covariate in the model the pattern of findings remained similar.

We repeated the RM-ANOVA for explicit ratings of actual and ideal body image. We found a significant main effect of body dissatisfaction, *F*(1, 64) = 57.29, *p* < .001, *η*^2^ = .47 BF_10_ = 8.440e + 16, and explicit RRT ratings, *F*(1, 64) = 188.01, *p* < .001, *η*^2^ = .75, BF_10_ = 490458.89, as well as a significant interaction between body dissatisfaction and explicit RRT ratings, *F*(1, 64) = 14.48, *p* < .001, *η*^2^ = .19, BF_10_ = 67.50. T-tests showed that individuals high in body dissatisfaction scored, on average, higher on actual body image ratings than individuals low in body dissatisfaction, *t*(48.92) = 7.05, *p* < .001, *d* = 1.79, BF_10_ = 1.608e + 07. This indicates that individuals high in body dissatisfaction were more likely to agree with statements referring to “I am fat/I am not thin” than individuals low in body dissatisfaction. In addition, we found that individuals high in body dissatisfaction scored, on average, higher on ideal body image ratings than individuals low in body dissatisfaction, *t*(64) = 5.53, *p* < .001, *d* = 1.36, BF_10_ = 20250.25. The latter indicates that individuals high in body dissatisfaction were more likely to agree with statements referring to “I want to be thinner/I do not want to be fatter” than individuals low in body dissatisfaction.

### Are Implicit Measures of Body Image and Their Interaction Related to Self-Reported Body Dissatisfaction Over and Above Explicit Measures?

**Fig. 1:**
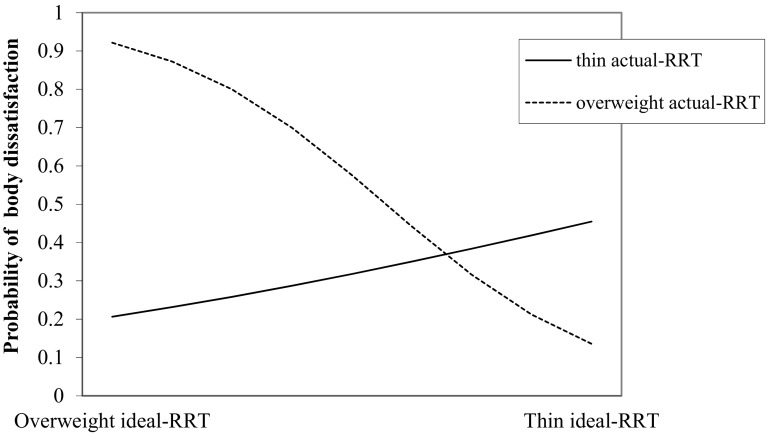
Probability of group membership (high vs. low degree of body dissatisfaction) as a function of the implicit desire to possess a thin body (ideal-RRT), separated for thin actual body image (1 SD below average on actual-RRT) and overweight actual body image (1 SD above average on actual-RRT). *RRT* Relational Responding Task

Table 4 shows the outcomes of the hierarchical logistic regression analysis with body dissatisfaction (high vs. low) as dependent variable. Predictor variables were standardized. Odds ratios above 1 indicate a higher chance of belonging to the group high in body dissatisfaction. Results showed that explicit ratings of actual body image are more strongly related to body dissatisfaction than explicit ratings of ideal body image. Actual and ideal RRT scores did not explain additional variance over and above explicit ratings. Yet, the interaction between actual-RRT and ideal-RRT scores did contribute significantly to the model in the final step. To interpret these findings, we plotted the probabilities for the interaction term in Fig. 1. Unexpectedly, the figure indicates that, specifically for individuals high in actual-RRT scores (with relatively strong implicit I-am-fat beliefs), stronger implicit I-want-to-be-thinner beliefs are related to a *lower* probability of belonging to the body dissatisfied group. When BMI was included as predictor in the first step, the pattern of results remained the same.

**Table 4:** Summary of hierarchical logistic regression analysis for predicting body dissatisfaction (1 = high, 0 = low;* n*  = 66)

Predictor	Wald	OR	*χ* ^2^_*change*_
Step 1			39.53**
Explicit actual	10.19**	5.92	
Explicit ideal	2.99†	4.19	
Step 2			4.58
Explicit actual	6.61*	4.79	
Explicit ideal	3.70†	6.25	
Actual-RRT	.00	.97	
Ideal-RRT	4.01*	.07	
Step 3			5.71*
Explicit actual	6.11*	5.32	
Explicit ideal	4.73*	11.42	
Actual-RRT	.68	3.98	
Ideal-RRT	2.61	.11	
Actual-RRT × ideal-RRT	4.86*	.00^a^	

*RRT* Relational Responding Task

†*p* < .10; *p < .05; **p < .01

^a^Since odds ratios are not standardized, in this case the multiplication of two small values (interaction term) could result in a very small yet significant odds ratio

### Do Implicit Measures of Actual and Ideal Body Image Relate to Dietary Restraint?

Table 5 shows the outcomes of the hierarchical regression analyses with scores on EDE-Q restraint and self-reported food intake as dependent variables. Again, predictor variables were standardized. Table 6 shows the outcomes of the hierarchical logistic regression analysis with the behavioral measure (candy bar vs. no candy bar) as dependent variable. Results showed that explicit actual and ideal body image both showed an independent association with self-reported restraint. The stronger the I-am-fat beliefs and the stronger the I-want-to-be-thinner beliefs, the higher the self-reported restraint appeared to be. In addition, explicit actual and ideal body image jointly predicted self-reported caloric intake. Stronger I-am-fat beliefs together with I-want-to-be-thinner beliefs were related to a lower amount of caloric intake, although neither showed independent predictive validity. Then, explicit actual body image, but not ideal body image predicted a higher chance of not choosing a candy bar. Actual-RRT, ideal-RRT, and their interaction effect did not show additional predictive value for any of the dieting indices. When BMI was included as predictor in the first step, the pattern of results remained the same for EDE-Q restraint. However, for self-reported food intake and the behavioral measure, explicit ratings no longer showed predictive validity over and above BMI.

**Table 5:** Summary of hierarchical regression analysis for predicting EDE-Q restraint and self-reported caloric intake

Predictor	EDE-Q restraint (*n* = 66)				Self-reported food intake (*n* = 55)			
	*B*	*β*	*t*	*R* ^2^_*change*_	*B*	*β*	*t*	*R* ^2^_*change*_
Step 1				.45**				.12*
Explicit actual	.38	.30	2.35*		− 133.59	− .27	− 1.46	
Explicit ideal	.83	.42	3.29**		− 67.08	− .09	− .49	
Step 2				.00				.01
Explicit actual	.39	.31	2.18*		− 124.62	− .25	− 1.28	
Explicit ideal	.84	.43	3.18**		− 45.26	− .06	− .32	
Actual-RRT	− .03	− .02	− .02		− 28.69	− .06	− .39	
Ideal-RRT	− .02	− .01	− .14		− 38.03	− .08	− .57	
Step 3				.01				.03^a^
Explicit actual	.37	.30	2.11*		− 118.55	− 1.23	− .25	
Explicit ideal	.84	.43	3.19**		− 46.52	− .33	− .42	
Actual-RRT	.04	.03	.27		− 61.97	− .79	− .81	
Ideal-RRT	.00	.00	.02		− 54.80	− .81	− .77	
Actual-RRT × ideal-RRT	− .17	− .12	− 1.18		100.38	1.26	1.16	

*EDE-Q* Eating Disorder Examination Questionnaire, *RRT* Relational Responding Task

*p < .05; **p < .01

^a^When conducting the analysis with the raw-score of the RRT, this step was significant (*R*^2^_*change*_ = .09)

**Table 6:** Summary of hierarchical logistic regression analysis for predicting behavioral measure of food selection: candy bar (= 0) versus no candy bar (= 1) (*n* = 66)

Predictor	Wald	OR	*χ* ^2^_*change*_
Step 1			8.95*
Explicit actual	2.68	2.05	
Explicit ideal	.62	1.63	
Step 2			2.34
Explicit actual	4.04	2.72*	
Explicit ideal	.75	1.74	
Actual-RRT	2.05	.19	
Ideal-RRT	.11	1.38	
Step 3			.17
Explicit actual	4.11	2.78*	
Explicit ideal	.75	1.75	
Actual-RRT	2.19	.16	
Ideal-RRT	.07	1.29	
Actual-RRT × ideal-RRT	.17	4.71	

*RRT* Relational Responding Task

*p < .05; **p < .01

## Discussion

The aim of this study was to examine the relation between self-reported body dissatisfaction and implicit actual and ideal body image beliefs and whether such implicit beliefs are related to dieting behaviors. In line with prior findings (Heider et al. 2015, 2018), results showed that women high in body dissatisfaction scored generally higher on implicit *actual* body image than individuals low in body dissatisfaction. However, both groups did not differ significantly in terms of implicit *ideal* body image. The latter finding is in contrast with the findings of Heider et al. (2015, 2018) who found that women high in body dissatisfaction displayed stronger implicit I-want-to-be-thinner beliefs than individuals low in body dissatisfaction. Although implicit measures of actual and ideal body image did not add to the prediction of self-reported body dissatisfaction over and above explicit measures of actual and ideal body image (cf. Heider et al. 2015, 2018), the interaction between actual and ideal implicit body image did. Surprisingly, however, the nature of this relation was opposite to our hypothesis. That is, specifically for individuals with relatively strong implicit I-am-fat beliefs, stronger implicit I-want-to-be-thinner beliefs were related to a lower probability of belonging to the body-dissatisfied group, whereas the pattern was reversed in individuals with relatively strong implicit I-am-thin beliefs. Finally, also in contrast to our expectations, implicit body image was not related to any of the dieting indices. Present findings concerning implicit body image beliefs were independent of BMI.

At first sight, it seems hard to explain the unexpected findings for the ideal-RRT. The sample of the present study was very similar to the previous samples of Heider et al. (2015, 2018). All three studies investigated female university students of around the same age with a similar mean BMI who had scored within the first or fourth quartile on a screening instrument for negative body image. The only prominent methodological difference with Heider et al. (2018) was the mother tongue of the participants. However, post-hoc analyses did not show a main effect of language (Dutch vs. English) nor an interaction effect between language and group (high vs. low body dissatisfaction) on ideal-RRT scores. In addition, the explicit ratings of the RRT stimuli indicated that individuals high in body dissatisfaction scored higher on actual body image (I-am-fat beliefs) and ideal body image (I-want-to-be-thinner beliefs) than individuals low in body dissatisfaction. This finding is in line with the definition of body dissatisfaction as the difference between actual and ideal body image and thereby attests to the validity of the statements. Surprisingly, ideal-RRT scores correlated with practically none of the other variables. In contrast, the actual-RRT scores correlated positively with explicit actual body image, body dissatisfaction, explicit ideal body image, and BMI. Perhaps due to a higher complexity of the statements, the ideal-RRT was a more difficult task than the actual-RRT, resulting in more noise and a reduction of the sensitivity of the task. This would be in line with the observation that participants were, on average, slower to respond in the ideal-RRT (*M* = 1860 ms) than in the actual-RRT (*M* = 1504 ms). In this respect, it should also be noted, that the power in the present study for the post-hoc t-tests of the RM-ANOVAs was suboptimal (power = .55 to detect a medium effect). Additional Bayesian analyses further showed that with respect to the ideal-RRT data (unlike the actual-RRT data) there was insufficient evidence to either confirm or reject the null hypothesis.

Our second goal was to examine whether implicit body image relates to dieting behavior. Results showed that implicit actual and ideal body image as well as their interaction did not explain unique variance in dieting behavior above explicit actual and ideal body image. We did find that explicit actual and ideal body image were predictive of the EDE-Q restraint scores. Individuals with relatively strong explicit I-am-fat beliefs and relatively strong explicit I-want-to-be-thinner beliefs reported higher levels of restraint eating. Likewise, explicit actual and ideal body image were predictive of the amount of self-reported caloric intake and food selection although explicit ratings no longer showed predictive validity over and above BMI. However, on basis of these correlational data we cannot determine if and how BMI, explicit actual and ideal body image, and dieting behavior are causally related. Unless a Type-II error is at play, the RRT results suggest that indices of implicit actual and ideal body image are not related to dieting. As mentioned in the introduction, it is often argued that the influence of implicit processes on behavior increases as a function of the degree to which the behavior occurs under automaticity conditions (Gawronski and Bodenhausen 2006; Wilson 2002). Two measures used to capture dieting behaviors in our study, however, were self-report measures. One could thus argue that these outcome measures primarily reflect the operation of explicit rather than implicit processes. Our intention was to include the behavioral measure of food selection as a more spontaneous measure of dieting, which we expected to be related to implicit processes. However, the purpose behind this measure might have been too obvious to the participants, which might have decreased the influence of implicit processes for this measure. It might be worthwhile in future studies to try to measure more spontaneous eating behavior, for example in a bogus taste test (e.g., Descheemaeker et al. 2014). Alternatively, one could also argue that dieting behaviors (as opposed to the act of reporting these behaviors) are mainly driven by explicit processes. In line with such an argumentation, we observed that explicit body image indices were indeed predictive of the dieting measures. Considering that many people have a problem sticking to their diet, it seems importance to distinguish between the intention to diet and actual dieting behaviors or “success” (e.g., Stroebe et al. 2013). While the intention to diet might be driven by explicit body image beliefs, individuals’ success in keeping their diet might be explained by relatively automatic cognitions leading to spontaneous (unwanted) food choices.

Our results indicate that implicit indices of actual and ideal body image do not help to explain self-reported body dissatisfaction over and above explicit body image indices (apart from the fact that the interaction effect between implicit ideal and actual body image showed a small independent relation with self-reported body dissatisfaction in an unexpected direction). In addition, implicit body image beliefs were not associated with the current measures of dieting. Nevertheless, it seems premature to conclude on basis of the present data and design that implicit body image indices are irrelevant in predicting cognitions and behavior in the body image domain. It could very well be that the present design was not optimal in testing the potential additive value of implicit measures. If explicit body image beliefs are in line with one’s dieting intentions, as probably was the case in our sample, implicit measures might not add much in explaining body dissatisfaction and dieting. However, when someone wants to change explicit beliefs, for example in therapy, implicit beliefs might become more important in explaining why some people successfully recover and others do not. Implicit beliefs might also be predictive of relapse after successful treatment. The latter is in line with some studies showing that treatment-induced changes in explicit cognitions not necessarily go together with changes in implicit cognitions (e.g., Huijding and Jong 2009), and residual implicit cognitions predicted post-treatment return of symptoms (e.g., Spruyt et al. 2015; Vasey et al. 2012). Therefore, it seems important to follow-up the present study with a sample of participants who would like to develop a more positive body image and/or try to stop dieting. This gives the possibility to investigate whether implicit indices of body image beliefs add predictive value in explaining who is successful and who is not, and in addition whether residual implicit body image beliefs after treatment are predictive of relapse.

Although the present study has several strengths, such as the use of relational tasks to measure both implicit actual and ideal body image beliefs in one sample, there are also some limitations that should be considered when interpreting these results. First of all, it should be mentioned that the sample size was relatively small and that the drop-out was quite substantial for the food diary which might have been due to the effort and discipline it takes to fill in a food diary during three consecutive days. Furthermore, the use of a state-like measure of body dissatisfaction (i.e., the BISS) might have been suboptimal because of potential fluctuations in body dissatisfaction over time. However, considering that body dissatisfaction typically remains stable over time (e.g. Tiggemann 2004), the use of a state measure of body dissatisfaction might still be viewed as a reasonable proxy of trait body dissatisfaction. Nevertheless, future studies could perhaps better use trait-like measures of body dissatisfaction such as the Multidimensional Body-Self Relations Questionnaire (Brown et al. 1990). Finally, one may note that the internal consistency estimates of the two RRT measures (i.e., Rsb = .49 and Rsb = .45 for the Ideal-RRT and Actual-RRT, respectively) were in line with prior findings of Heider et al. (2018), but relatively modest in comparison with the internal consistency estimate of the RRT reported by De Houwer et al. (2015, Rsb = .64). At this point, we can only speculate about the reasons for this discrepancy. Possibly, differences between implicit beliefs in the body image domain are larger than differences between implicit beliefs in other domains, like in the domain of racial prejudice as examined in De Houwer et al. (2015). For example, the (implicit) wish to weigh less not necessarily goes together with the wish to have a skinny figure, while both statements are used in the present study. In the internal consistency estimates, these items are treated as being interchangeable, potentially resulting in an underestimation of the reliability of the RRT. In other words, the modest internal consistency estimates observed in the present study could be an accurate reflection of the empirical truth rather than an indication of the limited reliability of the RRT. In line with this assertion, the internal consistency estimates reported by Heider et al. (2018), also in the domain of body dissatisfaction, were roughly in the same range as the ones reported here (i.e., Rsb = .57 and Rsb = .49 for the ideal-RRT and actual-RRT, respectively). Using the IRAP as a measure of implicit beliefs concerning body image, Heider et al. (2015), obtained even lower internal consistency estimates (i.e., Rsb = .24 and Rsb = .32 for the ideal-RRT and actual-RRT, respectively). For future studies, it could thus be worthwhile to look for ways to estimate internal consistency estimates at the level of specific items. However, it should be acknowledged that if indeed body image beliefs are relatively variable, this would still reduce the sensitivity of the current task as a measure of (implicit) body image beliefs.

## Conclusions

The present study showed that individuals with high body dissatisfaction are characterized by stronger I-am-fat beliefs than individuals low in body dissatisfaction, both at the explicit and the implicit level. Specifically, actual body image beliefs showed a strong and independent relation with body dissatisfaction. Implicit body image beliefs did not show independent predictive validity for body dissatisfaction over and above explicit body image beliefs. Finally, implicit body image was not related to dieting. Stronger explicit I-am-fat beliefs and I-want-to-be-thinner beliefs predicted more self-reported restraint eating and dieting behavior. Based on this cross-sectional design, we should be cautious with clinical implications and statements referring to causality. Nevertheless, in the treatment of excessive dieting it could be relevant to address both actual and ideal body image beliefs, whereas in the treatment of body dissatisfaction it might be particularly relevant to address primarily actual body image beliefs.

## Appendix

See Tables 7, 8 and 9.

**Table 7:** Inducer stimuli of the Relational Responding Tasks

Dutch	English
Goed	Good
Juist	Right
Correct	Correct
Exact	Exact
In orde	Okay
Mis	Mistaken
Onjuist	False
Incorrect	Incorrect
Fout	Error
Verkeerd	Wrong

**Table 8:** Target statements of the actual-Relational Responding Task in Dutch and English

Dutch	English
Ik heb een tenger figuur	I have a thin figure
Ik weeg weinig	I weigh little
Ik zie mezelf als een slank persoon	I see myself as a slender person
Ik heb een mager figuur	I have a skinny figure
Ik ben fijngebouwd	I am petite
Ik heb geen zware lichaamsbouw	I am not heavily built
Ik weeg niet te veel	I do not weigh too much
Ik zie mezelf niet als een dik persoon	I do not see myself as a fat person
Ik heb geen mollig figuur	I do not have a chubby figure
Ik heb geen stevig figuur	I do not have a robust figure
Ik heb geen tenger figuur	I do not have a thin figure
Ik weeg niet te weinig	I do not weigh too little
Ik zie mezelf niet als een slank persoon	I do not see myself as a slender person
Ik heb geen mager figuur	I do not have a skinny figure
Ik ben niet fijngebouwd	I am not petite
Ik heb een zware lichaamsbouw	I am heavily built
Ik weeg te veel	I weigh too much
Ik zie mezelf als een dik persoon	I see myself as a fat person
Ik heb een mollig figuur	I have a chubby figure
Ik heb een stevig figuur	I have a robust figure

**Table 9:** Target statements of the ideal-Relational Responding Task in Dutch and English

Dutch	English
Ik wil een meer tenger figuur hebben	I want to have a thinner figure
Ik streef ernaar minder te wegen	I strive to weigh less
Het is mijn wens om slanker te zijn	It is my wish to be more slender
Ik wil een mager figuur hebben	I want to have a skinny figure
Ik wil fijner gebouwd zijn	I want to be more petite
Ik wil een minder zware lichaamsbouw hebben	I want to be less heavily built
Ik streef er niet naar meer te wegen	I do not strive to weigh more
Het is niet mijn wens om dikker te zijn	It is not my wish to be fatter
Ik wil geen mollig figuur hebben	I do not want to have a chubby figure
Ik wil een minder stevig figuur hebben	I want to have a less robust figure
Ik wil een minder tenger figuur hebben	I want to have a less thin figure
Ik streef er niet naar minder te wegen	I do not strive to weigh less
Het is mijn wens om minder slank te zijn	It is my wish to be less slender
Ik wil geen mager figuur hebben	I do not want to have a skinny figure
Ik wil minder fijngebouwd zijn	I want to be less petite
Ik wil een zwaardere lichaamsbouw hebben	I want to be more heavily built
Ik streef ernaar meer te wegen	I strive to weigh more
Het is mijn wens om dikker te zijn	It is my wish to be fatter
Ik wil een mollig figuur hebben	I want to have a chubby figure
Ik wil een steviger figuur hebben	I want to have a more robust figure

## Footnotes

1 Because there is still debate about the best way to handle reaction time data, we repeated the analyses without dividing the difference in mean response latency between the two blocks by the pooled *SD* (raw-score; Blanton et al. 2015). When outcomes differ markedly from the original D-scores, we report this in a footnote.
